# Matter–wave interference and deflection of tripeptides decorated with fluorinated alkyl chains

**DOI:** 10.1002/jms.4514

**Published:** 2020-05-04

**Authors:** Jonas Schätti, Valentin Köhler, Marcel Mayor, Yaakov Y. Fein, Philipp Geyer, Lukas Mairhofer, Stefan Gerlich, Markus Arndt

**Affiliations:** ^1^ Faculty of Physics University of Vienna Boltzmanngasse 5, 1090 Vienna Austria; ^2^ Department of Chemistry University of Basel CH‐St. Johannsring 1 Basel 4056 Switzerland; ^3^ Institute of Nanotechnology Karlsruhe Institute of Technology Hermann‐von‐Helmholtz‐Platz 1, 76344 Eggenstein‐Leopoldshafen Germany; ^4^ Lehn Institute of Functional Materials (LIFM) Sun Yat‐Sen University (SYSU) XinGangXi Rd. 135, 510275 Guangzhou China

**Keywords:** deflectometry, matter‐waves, molecular beams, molecule interferometry, tripeptides

## Abstract

Studies of neutral biomolecules in the gas phase allow for the study of molecular properties in the absence of solvent and charge effects, thus complementing spectroscopic and analytical methods in solution or in ion traps. Some properties, such as the static electronic susceptibility, are best accessed in experiments that act on the motion of the neutral molecules in an electric field. Here, we screen seven peptides for their thermal stability and electron impact ionizability. We identify two tripeptides as sufficiently volatile and thermostable to be evaporated and interfered in the long‐baseline universal matter‐wave interferometer. Monitoring the deflection of the interferometric molecular nanopattern in a tailored external electric field allows us to measure the static molecular susceptibility of Ala–Trp–Ala and Ala–Ala–Trp bearing fluorinated alkyl chains at C‐ and N‐termini. The respective values are $4\pi \varepsilon_{0}\times \left(330\pm 150\right)\;{Å}^{3}$ and $4\pi \varepsilon_{0}\times \left(270\pm 80\right)\;{Å}^{3}$.

## 1 MAIN TEXT

While analytical chemistry is a mature and progressive field that enables all molecular sciences, analytical experiments on isolated neutral biomolecules are scarce. The work presented here is part of a research program aimed at developing a set of tools for the exploration of biomolecules in the gas phase in a controlled local environment.^1^, ^2^, ^3^ We focus in particular on molecule interferometry in the center of mass motion, where we exploit the quantum wave nature of matter to form interference fringes in the density of a molecular beam in free flight. In intense molecular beams, the spatial phase of these fringes can be read out with nanometer accuracy and thus provides high sensitivity to external forces acting on the particles.^4^ Interferometric measurements of electronic, magnetic, and optical properties of neutral particle beams thus become accessible.^5^, ^6^, ^7^, ^8^, ^9^, ^10^

As in Otto Stern's experiments a century ago,^11^ a key challenge lies in the preparation and detection of molecular beams, even more so for fragile biomolecules.

A well‐established technique in physical chemistry is to employ short‐pulse laser desorption of analyte molecules into a supersonically expanding noble gas beam.^12^, ^13^, ^14^, ^15^, ^16^ This has been successfully employed in peptide deflectometry to measure the electric susceptibility of various small peptides, also as a function of amino acid sequence.^2^, ^17^, ^18^, ^19^, ^20^, ^21^

Recent experiments have shown that ultrafast laser desorption can successfully launch fluoroalkyl‐functionalized Trp–Lys polypeptides of up to 50 amino acid residues in length and 20 000 amu in mass,^22^ and have even shown matter‐wave interference of a peptide composed of 15 amino acids.^23^

In contrast to supersonic expansions, thermal beams can provide high beam density, not limited by the duty cycle of a pulsed experiment. Thermal sources also produce molecular beams with 60% lower velocities than their supersonic counterparts. This can be an advantage in beam deflection and matter‐wave experiments.

We focus here on thermal beams of biomolecules, which typically exhibit a strong tendency to fragment at the weakest intramolecular bond. Despite their fragility, thermal beams of nucleotides^24^ and individual amino acids^25^ have been prepared successfully for mass spectrometry before. It has also been recently demonstrated^26^, ^27^, ^28^ that highly fluorinated alkyl tags can facilitate molecular volatilization. It is interesting to note that fluorination of biomolecules is a frequently used technique to enhance the stability of proteins against chemical and thermal denaturation, while maintaining the molecule's structure and biological activity.^29^ Here, we use a thermal beam of functionalized tripeptides for quantum interference experiments and quantum‐assisted electric deflection measurements.

## 2 MOLECULAR DESIGN, SYNTHESIS, AND CHARACTERIZATION

Because intact evaporation of unprotected amino acids or functional peptides has been proven to be challenging in previous experiments,^28^ we focus our present study on seven tailored tripeptides that we designed, synthesized, and analyzed in near‐field matter‐wave interferometry, starting from an effusive beam.

The goal was to investigate the role of the length of the protecting fluoroalkyl tags and to identify sufficiently stable and volatile molecules for use in matter‐wave interferometry. Different sequences of Trp–Ala–Ala, Trp–Gly–Lys, Tyr–Trp–Gly, and Trp–Pro–Ala were equipped with fluorinated alkyl chains at C‐ and N‐termini to study their behavior in the evaporation and ionization experiment (see Supporting Information for structural details).

Peptides carrying the N‐terminal fluoroalkyl tag were synthesized by standard solid phase peptide synthesis on chlorotrityl resin. The fluoroalkyl chain at the C‐terminus was introduced after cleavage from the resin. Peptides with lysine residues were synthesized on a *Rink*‐amide support and modified by amidation with the NHS‐esters of the corresponding fluoroalkyl carboxylic acids.

**FIGURE 1:**
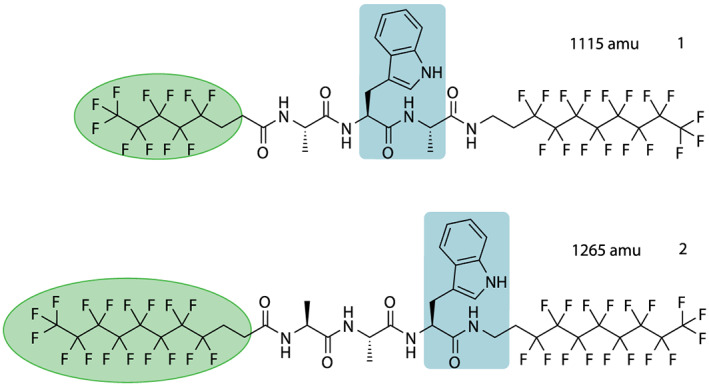
Tailored tripeptides that successfully showed matter‐wave interference. Among the tested compounds (see Supporting Information), **1** and **2** proved the most compatible for use in a long‐baseline molecule interferometer [Colour figure can be viewed at wileyonlinelibrary.com]

We find that among all model systems (**1**–**7**), only **1** and **2** (Figure 1) could be evaporated and detected in electron impact ionization quadrupole mass spectrometry with sufficient intensity and stability for subsequent quantum interference experiments. Compounds **3**–**7** (see Supporting Information) showed excessive fragmentation either due to the long flight distance in our interferometer setting, the subsequent harsh electron impact ionization, or possibly a combination of both.^28^ Several volatilization techniques were tested, including pure thermal evaporation and expansion of a thermal cloud into a noble gas, as well as direct laser desorption without matrix or seed gas. Of the tested methods, pure thermal beams provided the most reliable source for these experiments.

## 3 QUANTUM INTERFERENCE AND ELECTRIC SUSCEPTIBILITY

**FIGURE 2:**
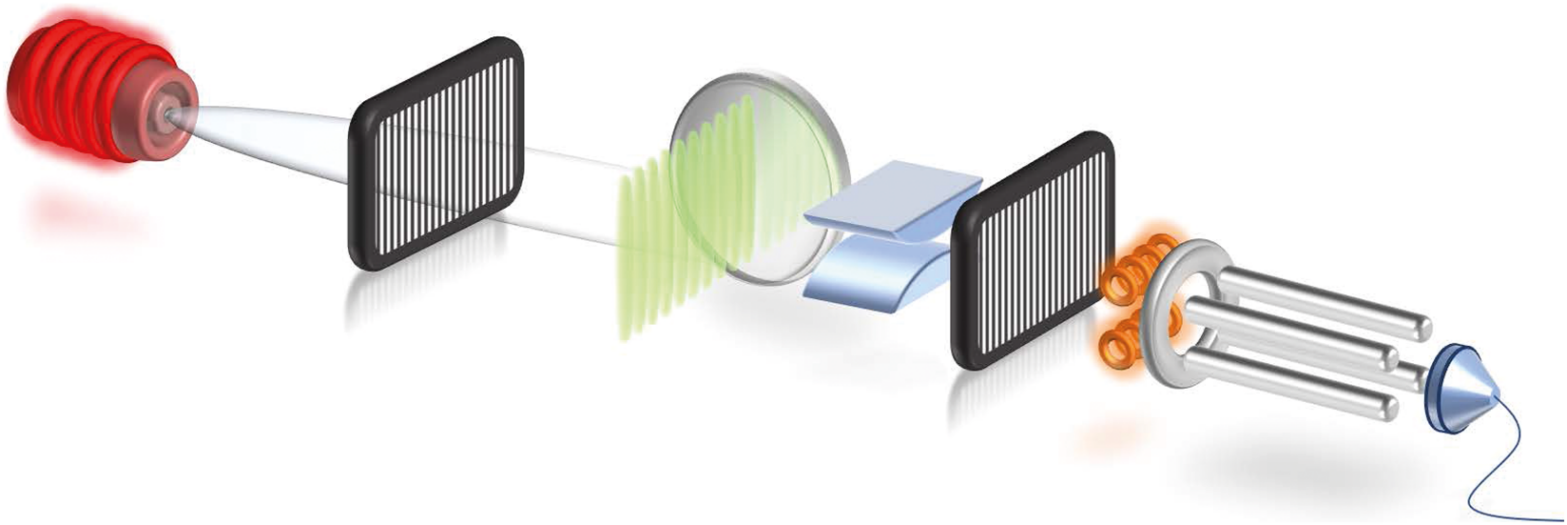
Long baseline molecule interferometer and electric deflectometer. The peptides are evaporated in an oven (left), pass the three gratings (G_1_–G_3_), and are ionized by electron impact and mass‐selected in a quadrupole mass spectrometer (right). G_1_ and G_3_ are 266‐nm transmission gratings implemented as nanofabricated structures in silicon nitride, whereas G_3_ is an optical phase grating implemented as a standing wave of 532‐nm light. Diffraction in G_1_ prepares transverse coherence to cover several nodes of the standing light wave, which forms G_2_, which satisfies the necessary interference condition to have at least two fundamentally indistinguishable semiclassical paths. The electrode after G_2_ imprints a position and velocity‐dependent phase, which shifts the interference pattern at the position of G_3_. We detect the interference pattern by shifting G_3_ transversely and counting the transmitted molecules

The molecules are evaporated in a resistively heated oven and pass three gratings, each with a 266‐nm period and a 1‐m separation between them (Figure 2).

The first and third gratings (G_1_ and G_3_) are nanomechanical silicon nitride structures, whereas the second grating (G_2_) is formed by a standing wave of green light. The molecules have a Gaussian velocity distribution with center velocities of *v* = 140–150 m/s and spreads of 18–21% of the mean velocity, with de Broglie wavelengths of $\lambda_{\mathrm{db}}=\frac{h}{\mathrm{mv}}=$ 3–5 pm. When arriving at G_1_, the molecules do not have any significant transverse coherence. Quantum delocalization in the molecules' center of mass motion emerges as a result of the Heisenberg uncertainty principle. Confinement of the molecules as they pass through the grating openings creates a momentum uncertainty that results in a position uncertainty of more than the separation of two antinodes in the green standing light wave. Because each tripeptide has more than two fundamentally indistinguishable pathways through the second grating to arrive at the final grating G_3_, the wave amplitudes associated with these trajectories can interfere. This interference presents itself as a density pattern with 266 nm periodicity at the position of G_3_. We detect this by scanning G_3_ transversely across the molecular beam and counting the transmitted molecules in a quadrupole mass spectrometer. Recent experiments have shown that even molecules in excess of 25 000 amu exhibit interference in this setup.^30^

**FIGURE 3:**
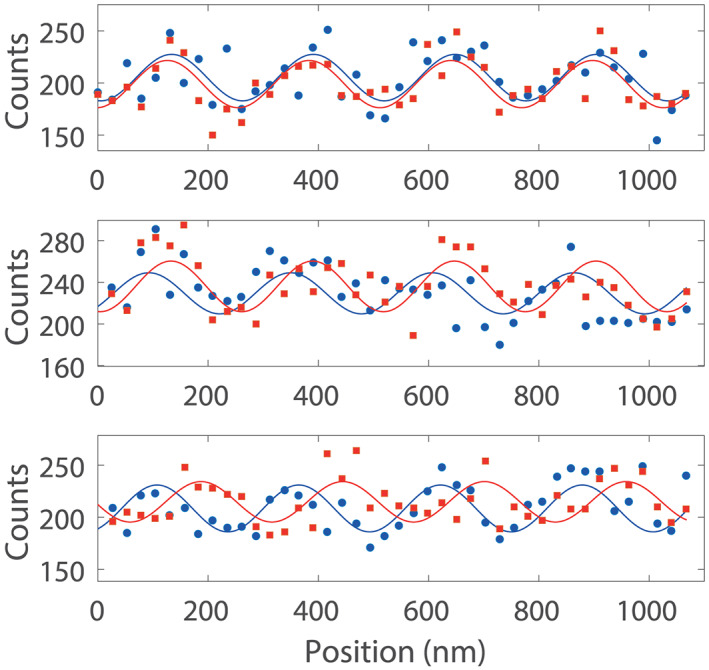
Interferometric deflection of Compound **1**. Scanning G_3_ over the molecular beam and counting the molecules reveal interference as a sinusoidal modulation of the transmitted flux. (A)–(C) shows the increasing deflection for 250 V (undeflected with respect to the reference at the same voltage), 500 V, and 750 V, respectively. Blue circles indicate interference scans with a reference voltage at 250 V. Red squares show data with taken deflection voltage. The data contains an unsubtracted dark rate of 15 counts/s, with each point integrated for 3 s

Interference and deflection data for Compound **1** is shown in Figure 3. The present experiments are limited by the molecular beam intensity, detection efficiency, and signal‐to‐noise ratio. The interference fringes are shifted transversely by an amount Δ*x* ∝ *χ*_*e*_(***E*** · ∇)*E*_*x*_/*mv*^2^ in the presence of a tailored electric field with a constant and homogeneous value of (***E*** · **∇**)*E*_*x*_ **.** Here, *χ*_*e*_ = *α*_stat_+〈*d*^2^ 〉_*T*_/3*kT* is the molecule's electric susceptibility, which includes the static polarizability *α*_stat_ and the temperature‐dependent vibrationally induced average of an electric dipole *d*, which is relevant in even nonpolar floppy molecules.^8^, ^31^, ^32^ A permanent dipole moment generally does not lead to fringe shifts but to a loss of fringe contrast, due to the random initial molecular orientation and rotation. The temperature *T* can be assumed to be the temperature of the source, 275–290°C in these experiments. Details on the extraction of susceptibilities in such a setup and the calibration of the electrode with atomic cesium are discussed in Fein et al.^33^

**FIGURE 4:**
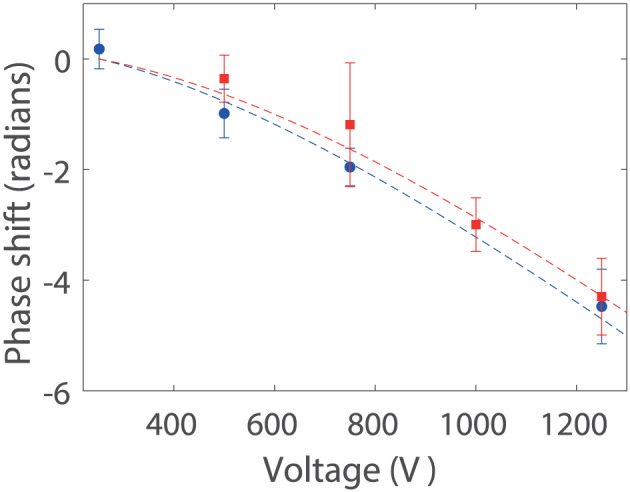
Matter‐wave assisted Stark deflectometry. The interference fringes shift laterally when the homogeneous (***E*** · **∇**)***E***_***x***_ field is increased by ramping up the deflection voltage. From the quadratic dependence of the fringe position on electrode voltage, we derive the susceptibility ***χ***. The error bars represent 68% confidence intervals of the fitted phases. Blue circles are Compound **1**, and red squares are Compound **2** [Colour figure can be viewed at wileyonlinelibrary.com]

The deflection data for Compounds **1** and **2** are shown in Figure 4. Each single interference scan is composed of 42 position steps of 26 nm, integrated for 3 s per point. By varying the electrode voltage and tracking the phase of the deflected interference fringes, we can extract $\chi_{1}=4\pi \varepsilon_{0}\times \left(330\pm 150\right)\;{Å}^{3}$ and $\chi_{2}=4\pi \varepsilon_{0}\times \left(270\pm 80\right)\;{Å}^{3}$ for Compounds **1** and **2**, respectively. The error bars contain the statistical uncertainty and the systematic uncertainty in the velocity distribution added in quadrature. The susceptibilities of the two molecules are similar within their error bars and consistent with expectations based on previous classical deflection studies of related compounds.^2^ The resolution in these experiments does not yet allow us to discriminate structurally related thermal tripeptides based on their susceptibility. However, previous classical beam deflection experiments with tripeptides composed of glycine, tryptophan, and tyrosine^2^ show that sequence isomers can differ in susceptibility by up to $4\pi \varepsilon_{0}\times 100\;{Å}^{3}$, indicating that the technique described here can be used to discriminate isomers. This would require an improved signal‐to‐noise ratio which may be achievable with improved source techniques.

## 4 CONCLUSION AND OUTLOOK

We have demonstrated the feasibility to observe matter–wave interference and extract electric susceptibility values of a thermal beam of functionalized tripeptides. Future experiments with larger peptides, proteins, or DNA will profit from the same interference concept, where the molecular density pattern serves as a tool for measuring internal particle properties. Our study also shows that future experiments would benefit from cold sources for improved molecule stability and laser ionization detection for improved detection efficiency. Recent progress in ultrafast laser desorption and vacuum ultraviolet photoionization of tryptophan‐rich polypeptides,^22^ as well as progress in the neutralization of biomolecular beams prepared by electrospray ionization and photo‐cleavage neutralization,^34^, ^35^ are expected to enable research with a wide range of large peptides.^23^

## Supporting information


**Figure S1**. The five additional peptides tested in this study.
**Figure S2**. ^1^H‐NMR (DMSO‐*d*6, 500 MHz, 293 K) of compound **5**.
**Figure S3**. UPLC Chromatogram of compound **5**.
**Figure S4**. ^1^H ‐NMR (DMSO‐*d*6, 500 MHz, 293 K) of compound **6**.
**Figure S5**. UPLC Chromatogram of compound **6**.
**Figure S6**. ^1^H ‐NMR (DMSO‐*d*6, 500 MHz, 293 K) of compound **7.** HOAt impurity is indicated.
**Figure S7**. UPLC Chromatogram of compound **7**. HOAt impurity is indicated.
