# Supplementary material for: Matter–wave interference and deflection of tripeptides decorated with fluorinated alkyl chains
Source: J Mass Spectrom. 2020 May 4;55(6):e4514. doi: 10.1002/jms.4514 (PMC7317408; doi:10.1002/jms.4514)
Supplement: Supplementary file 1 — Figure S1. The five additional peptides tested in this study. Figure S2. 1H‐NMR (DMSO‐d6, 500 MHz, 293 K) of compound 5. Figure S3. UPLC Chromatogram of compound 5. Figure S4. 1H ‐NMR (DMSO‐d6, 500 MHz, 293 K) of compound 6. Figure S5. UPLC Chromatogram of compound 6. Figure S6. 1H ‐NMR (DMSO‐d6, 500 MHz, 293 K) of compound 7. HOAt impurity is indicated. Figure S7. UPLC Chromatogram of compound 7. HOAt impurity is indicated. [file JMS-55-e4514-s001.pdf]

Supporting Information

Matter-wave interference and deflection of tripeptides decorated with fluorinated alkyl chains

Jonas Schätti[b], Valentin Köhler[b], Marcel Mayor[b,c,d], Yaakov Y. Fein[a], Philipp Geyer[a], Lukas Mairhofer[a], Stefan Gerlich[a], and Markus Arndt*[a]

[a] Yaakov Y. Fein, Dr. Stefan Gerlich, Dr. Philipp Geyer, Dr. Lukas Mairhofer, Prof. Dr. Markus Arndt, University of Vienna, Faculty of Physics, Boltzmanngasse 5, 1090 Vienna, Austria, markus.arndt@univie.ac.at

[b] Dr. Jonas Schätti, Dr. Valentin Köhler, Prof. Dr. Marcel Mayor, Department of Chemistry, University of Basel, CH-St. Johannsring 1, Basel 4056, Switzerland

[c] Karlsruhe Institute of Technology, Institute of Nanotechnology, Hermann-von-Helmholtz-Platz 1, 76344, Eggenstein-Leopoldshafen, Germany

[d] Lehn Institute of Functional Materials (LIFM), Sun Yat-Sen University (SYSU), XinGangXi Rd. 135, 510275 Guangzhou, P.R. China.

Table of contents

Synthesis.......... 2

General information.......... 2

Modified Trp-Gly-Lys **5**.......... 2

Modified Tyr-Trp-Gly **6**.......... 3

Modified Trp-Pro-Ala **7**.......... 4

^{1}H-NMR spectra and LC traces.......... 6

Experimental details for interference and deflection experiments.......... 9

References.......... 9

Synthesis

General Information

Chemicals were purchased from Sigma Aldrich, Fluorochem, Apollo Scientific and used as received. UPLC-MS experiments were performed with an Acquity UPLC-H Class Bio from Waters equipped with a PDA and an SQ detector 2 with the following column: ACQUITY UPLC, HSS T3 1.8 µm, 2.1 x 100 mm. Solvents were water and acetonitrile, respectively, each containing 0.1% formic acid, later on, referred to as (A) and (B). The flow rate was set to 0.61 mL/min and the column temperature to 40 °C. The gradient of the Method was: 0 min – 90% A; 1 min – 90% A; 4 min – 100% B; 7.0 min – 100% B. Mass detection was performed in scan mode for positive ions (cone voltage 40 V, desolvation temperature: 600°C). NMR experiments were performed at 20°C on Bruker Avance III NMR spectrometers operating at 500 MHz proton frequency .The NMR spectrometer was equipped with inverse dual-channel broadband probe heads with z-gradients. ^{13}C shifts were determined by 2D NMR experiments (HMBC and HMQC). ^{1}H and ^{13}C signals were assigned by 2D NMR (COSY, HMBC, HMQC). All NMR spectra were recorded in DMSO-d^{6}.

The Synthesis of Peptides **1**-**4** is described elsewhere.[1]

Chemical Formula: $C_{38}H_{29}F_{34}N_5O_4$
Molecular Weight: 1265.6267

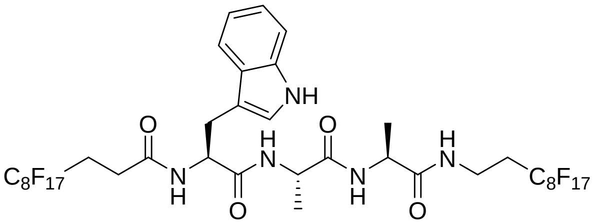

3

Chemical Formula: $C_{38}H_{29}F_{34}N_5O_4$
Molecular Weight: 1265.6267

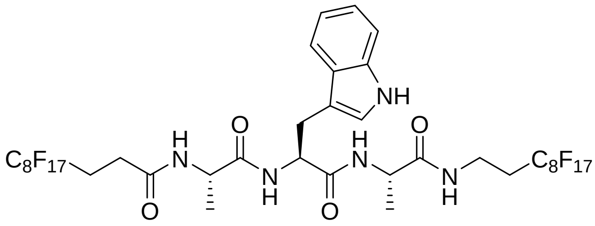

4

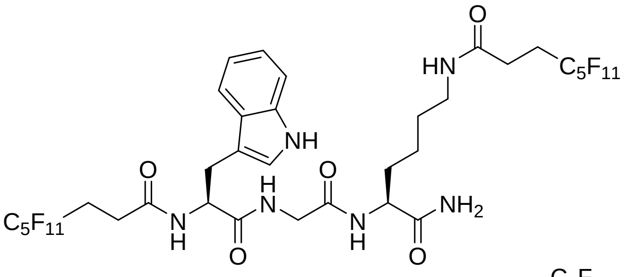

5

Chemical Formula: $C_{35}H_{34}F_{22}N_6O_5$
Molecular Weight: 1036.6589

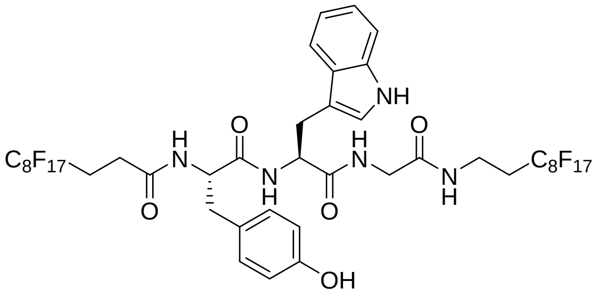

6

Chemical Formula: $C_{43}H_{31}F_{34}N_5O_5$
Molecular Weight: 1343.6967

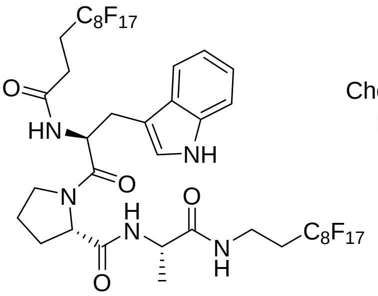

7

Chemical Formula: $C_{40}H_{31}F_{34}N_5O_4$
Molecular Weight: 1291.6647

Figure S1. The five additional peptides tested in this study.

Modified Trp-Gly-Lys **5**

The unmodified Trp-Gly-Lys was synthesized on a rink-amid resin (2.7 g, 0.54 mmol/g) according to previously published procedures.[1] The protected amino acids (3.00 eq., 4.37 mmol), PyBOP (3.00 eq., 2.27 g, 4.37 mmol) and DIPEA (6.00 eq., 1.45 mL, 8.74 mmol) were reacted for 2 h per coupling step. After cleavage from the resin with TFA and precipitation with Et_2O, the unmodified crude peptide (398 mg, 1.02 mmol, 70%) was obtained. It was directly dissolved in DMF (50 mL) and coupled to the NHS-ester of perfluoro-2*H*,2*H*,3*H*,3*H*-undecanoic acid (4.00 eq., 385 mg, 4.08 mmol) under stirring for 5 h at room temperature. The solvent was reduced to 20 mL, and water (20 mL) was added. The crude precipitate was reprecipitated twice from DMF by addition of water. The modified tripeptide **5** was obtained as a slightly orange powder (465 mg, 449 µmol, 44%). UPLC-MS: T_R = 4.29 min; MS (ESI+) m/z: 1037.5 [100%, M + H^+].

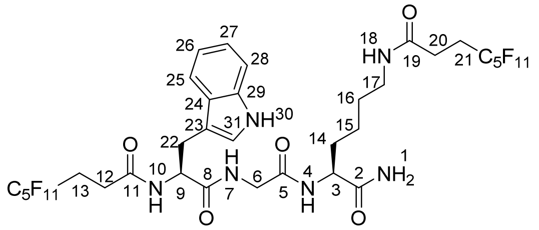

5

^{1}H-NMR (DMSO-d_6, 500 MHz, 293 K) δ: 10.91 (d, $^3J_{H-H}$ = 2.4 Hz, 1 H, H-30), 8.78 (d, $^3J_{H-H}$ = 7.8 Hz, 1 H, H-4), 8.72 (d, $^3J_{H-H}$ = 7.7 Hz, 1 H, H-4), 8.38 (t, $^3J_{H-H}$ = 5.9 Hz, 1 H, H-7), 8.19 (t, $^3J_{H-H}$ = 5.6 Hz, 1 H, H-18), 7.53 (d, $^3J_{H-H}$ = 7.9 Hz, 1 H, H-28), 7.30 (d, $^3J_{H-H}$ = 8.1 Hz, 1 H, H-25), 7.25 (s, 1 H, H-1), 7.14 (d, $^3J_{H-H}$ = 2.3 Hz, 1 H, H-31), 7.07 (s, 1 H, H-1), 7.03 (t, $^3J_{H-H}$ = 7.6 Hz, 1 H, H-26), 6.94 (t, $^3J_{H-H}$ = 7.4 Hz, 1 H, H-27),4.46-4.40 (m, 1 H, H-9), 4.18-4.11 (m, 1 H, H-3), 3.66 (dd, $^2J_{H-H}$ = 16.8 Hz, $^3J_{H-H}$ = 6.2 Hz, 1 H, H-6), 3.51 (dd, $^2J_{H-H}$ = 16.8 Hz, $^3J_{H-H}$ = 5.4 Hz, 1 H, H-6), 3.16 (dd, $^2J_{H-H}$ = 14.7 Hz, $^3J_{H-H}$ = 4.9 Hz, 1 H, H-22), 3.05-2.91 (m, 3 H, H-17 and H-22), 2.47-2.25 (H-12, H-13, H-20 and H-21), 1.55-1.40 (m, 2 H, H-14), 1.36-1.07 (m, 4 H, H-15 and H-16). ^{13}C-NMR δ: 171.9 (C-11), 171.7 (C-8), 170.8 (C-2), 169.6 (C-5), 169.0 (C-19), 135.9 (C-29), 127.2 (C-24), 123.4 (C-31), 120.3 (C-26), 118.0 (C-28), 117.8 (C-27), 110.9 (C-25), 110.0 (C-23), 53.7 (C-9), 53.0 (C-3), 41.7 (C-6), 37.8 (C-17), 31.0 (C-14), 28.2 (C-16), 26.6 (C-22), 25.5 (C-12, C-13, C-20 and C-21), 22.2 (C-15) all fluorinated carbon atoms were not detected.

Modified Tyr-Trp-Gly **6**

The unmodified Tyr-Trp-Gly was synthesized on 2-chlorotrityl chloride resin (2.10 g, 1 mmol/g) following previously published procedures.[1] The protected amino acids (3.00 eq., 6.30 mmol), PyBOP (3.00 eq., 3.28 g, 6.30 mmol) and DIPEA (6.00 eq., 2.09 mL, 12.6 mmol) were reacted for 2 h per coupling step. Perfluoro-2*H*,2*H*,3*H*,3*H*-undecanoic acid (2.00 eq., 2.07 g, 4.20 mmol) was coupled to the N-terminus of the resin-bound tripeptide with PyBOP (2.00 eq., 2.19 g, 4.20 mmol) and DIPEA (4.00 eq., 1.40 mL, 8.40 mmol) overnight. Subsequently, the peptide was cleaved from the resin with TFA and precipitated from Et_2O yielding the crude N-terminal modified peptide (820 mg, 913 µmol, 43%). To a solution of this peptide in DMF (100 mL) perfluoro-1*H*,1*H*,2*H*,2*H*-decan-1-amine (3.00 eq., 1.18 g, 2.74 mmol), HATU (3.00 eq., 1.04 g, 2.74 mmol) and DIPEA (6.00 eq., 906 µl, 5.48 mmol) were added and the mixture was stirred overnight at room temperature. The solvent was reduced to 20 mL, and water (20 mL) was added. The crude precipitate was reprecipitated twice from DMF by addition of water. The modified tripeptide 6 was obtained as a slightly orange solid (932 mg, 694 µmol, 76%). UPLC-MS: T_R = 4.93 min; MS (ESI+) m/z: 1366.3 [100%, M + Na^+].

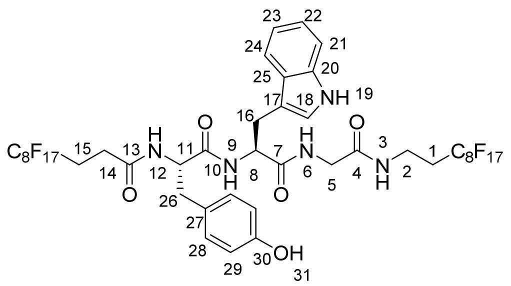

6

^{1}H-NMR (DMSO-d_6, 500 MHz, 293 K) δ: 10.84 (d, $^{3}J_{H-H}$ = 2.3 Hz, 1 H, H-19), 9.12 (s, 1 H, H-31), 8.20-8.11 (m, 3 H, H-6, H-9 and H-12), 7.87 (t, $^{3}J_{H-H}$ = 5.8 Hz, 1 H, H-3) 7.54 (d, $^{3}J_{H-H}$ = 7.9 Hz, 1 H, H-24), 7.32 (d, $^{3}J_{H-H}$ = 8.1 Hz, 1 H, H-21), 7.15 (d, $^{3}J_{H-H}$ = 2.3 Hz, 1 H, H-18), 7.06 (t, $^{3}J_{H-H}$ = 8.0 Hz, 1 H, H-22), 6.97 (t, $^{3}J_{H-H}$ = 8.1 Hz, 1 H, H-23), 6.89 (d, $^{3}J_{H-H}$ = 8.5 Hz, 2 H, H-28), 6.55 (d, $^{3}J_{H-H}$ = 8.5 Hz, 2 H, H-29), 4.43 (q, $^{3}J_{H-H}$ = 6.9 Hz, 1 H, H-8), 4.33-4.27 (m, 1 H, H-11), 3.78 (dd, $^{2}J_{H-H}$ = 16.7 Hz, $^{3}J_{H-H}$ = 6.4 Hz, 1 H, H-5), 3.50 (dd, $^{2}J_{H-H}$ = 16.7 Hz, $^{3}J_{H-H}$ = 5.1 Hz, 1 H, H-5), 3.33 (m, 2 H, H-2), 3.19 (dd, $^{2}J_{H-H}$ = 14.7 Hz, $^{3}J_{H-H}$ = 5.5 Hz, 1 H, H-16), 3.02 (dd, $^{2}J_{H-H}$ = 14.7 Hz, $^{3}J_{H-H}$ = 8.3 Hz, 1 H, H-16), 2.81 (dd, $^{2}J_{H-H}$ = 14.1 Hz, $^{3}J_{H-H}$ = 4.7 Hz, 1 H, H-26), 2.58 (dd, $^{2}J_{H-H}$ = 14.1 Hz, $^{3}J_{H-H}$ = 9.6 Hz, 1 H, H-26), 2.45-2.24 (m, 6 H, H-1, H-14 and H-15). ^{13}C-NMR δ: 171.5 (C-7), 169.2 (C-13), 168.7 (C-4), 155.6 (C-30), 135.9 (C-20), 129.6 (C-28), 127.4 (C-27), 127.2 (C-25), 123.1 (C-18), 120.5 (C-22), 118.0 (C-24), 117.8 (C-23), 114.5 (C-29), 111.0 (C-21), 109.6 (C-17), 54.7 (C-11), 53.6 (C-8), 41.8 (C-5), 36.0 (C-26), 30.5 (C-2), 29.3 (C-1), 26.6 (C-16), 25.5 (C-14 and C-15), C-10 and all fluorinated carbon atoms were not detected.

Modified Trp-Pro-Ala **7**

Tripeptide **7** was prepared following the procedure for peptide **6** above. The N-terminal modified peptide (697 mg, 823 µmol, 39%) was collected after precipitation and converted into **7**. The product was obtained after precipitation in moderate purity (455 mg, 352 µmol, 71%) and contained a contamination of HOAt. UPLC-MS: T_R = 5.15 min; MS (ESI+) m/z: 1314.5 [100%, M + Na^{+}].

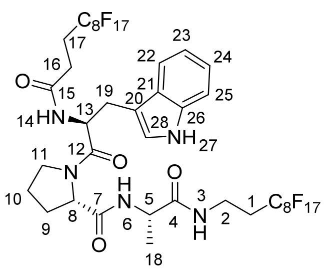

7

There are two rotamers present in solution indicated with R1 and R2 behind the atom number. ^{1}H-NMR (DMF-d_7, 500 MHz, 293 K) δ: 11.01 (d, $^{3}J_{H-H}$ = 2.4 Hz, 1 H, H-27, R1),), 10.93 (d, $^{3}J_{H-H}$ = 2.4 Hz, 1 H, H-27, R2), 8.71 (d, $^{3}J_{H-H}$ = 6.6 Hz, 1 H, H-14 , R1), 8.44 (d, $^{3}J_{H-H}$ = 7.5 Hz, 1 H, H-14, R2), 8.24 (d, $^{3}J_{H-H}$ = 7.5 Hz, 1 H, H-6, R1), 8.00 (d, $^{3}J_{H-H}$ = 7.3 Hz, 1 H, H-6 , R2), 7.97 (t, $^{3}J_{H-H}$ = 5.8 Hz, 1 H, H-3 , R2), 7.92 (t, $^{3}J_{H-H}$ = 5.9 Hz, 1 H, H-3 , R1),)), 7.66 (d, $^{3}J_{H-H}$ = 7.9 Hz, 1 H, H-22 , R2), 7.56 (d, $^{3}J_{H-H}$ = 7.9 Hz, 1 H, H-22 , R1), 7.43 (d, $^{3}J_{H-H}$ = 8.6 Hz, 1 H, H-25 , R1), 7.41 (d, $^{3}J_{H-H}$ = 8.2 Hz, 1 H, H-25 , R2), 7.35 (d, $^{3}J_{H-H}$ = 2.4 Hz, 1 H, H-28 , R2), 7.32 (d, $^{3}J_{H-H}$ = 2.4 Hz, 1 H, H-28 , R1), 7.14-7,07 (m, 1 H, H-24), 7.05-6.99 (m, 1 H, H-23), 4.97 (td, , $^{3}J_{H-H}$ = 7.9 Hz, $^{3}J_{H-H}$ = 5.1 Hz, 1 H, H-13, R2), 4.97 (dt, , $^{3}J_{H-H}$ = 8.9 Hz, $^{3}J_{H-H}$ = 6.5 Hz, 1 H, H-13, R1), 4.40 (dd, $^{3}J_{H-H}$ = 8.3 Hz, $^{3}J_{H-H}$ = 4.7 Hz, 1 H, H-8, R2), 4.37-4.22 (m, 1 H, H-5), 3.83

(dd, $^{3}J_{H-H}$ = 8.3 Hz, $^{3}J_{H-H}$ = 1.7 Hz, 1 H, H-8, R2), 3.79-3.73 (m, 1 H, H-11, R2), 3.66-3.58 (m, 1 H, H-11, R2), 3.57-3.44 (m, 1 H, H-2), 3.44-3.36 (m, 1 H, H-11, R1) 3.33-3.26 (m, 2 H, H-11, R1 and H-19, R2), 3.22-3.11 (m, 2 H, H-19, R1), 3.10-3.00 (m, 1 H, H-19, R2), 2.64-2.32 (m, 6 H, H-1, H-16 and H-17), 2.18-2.08 (m, 1 H, H-9 , R2), 2.01-1.93 (m, 2 H, H-9, R2 and H-10, R2), 1.89-1.81 (m, 1 H, H-10, R2), 1.78-1.70 (m, 1 H, H-9 , R1), 1.56-1.49 (m, 2 H, H-10 , R1), 1.35-1.30 (m, 3 H, H-18), 0.96-0.86 (m, 1 H, H-9, R1). ^{13}C-NMR δ: 172.7 (C-4), 171.7 (C-12, R2), 171.6 (C-7 , R2), 171.5 (C-7, R1), 171.2 (C-12, R1), 170.0 (C-15, R1, 169.8 (C-15, R2, 136.9 (C-26), 127.9 (C-21, R2), 127.6 (C-21, R1), 124.4 (C-28, R1), 124.1 (C-28, R2), 121.2 (C-24), 118.7 (C-22, R1), 118.6 (C-23), 118.3 (C-22, R2), 111.6 (C-25), 110.3 (C-20, R2), 109.6 (C-20, R1), 61.1 (C-8, R2), 60.5 (C-8, R1), 53.1 (C-13, R1), 52.6 (C-13, R2), 49.7 (C-5, R1), 49.2 (C-5, R2), 47.4 (C-11, R2), 46.5 (C-11, R1), 31.6 (C-2), 30.6 (C-9, R1), 30.2 (C-1), 28.9 (C-9, R2), 28.5 (C-19, R1), 27.3 (C-19, R2), 26.3 (C-16 and C-17), 24.9 (C-10, R2), 21.8 (C-10, R1), 17.4 (C-18), all fluorinated carbon atoms were not detected.

^{1}H-NMR spectra and LC traces for compounds **5** - **7**

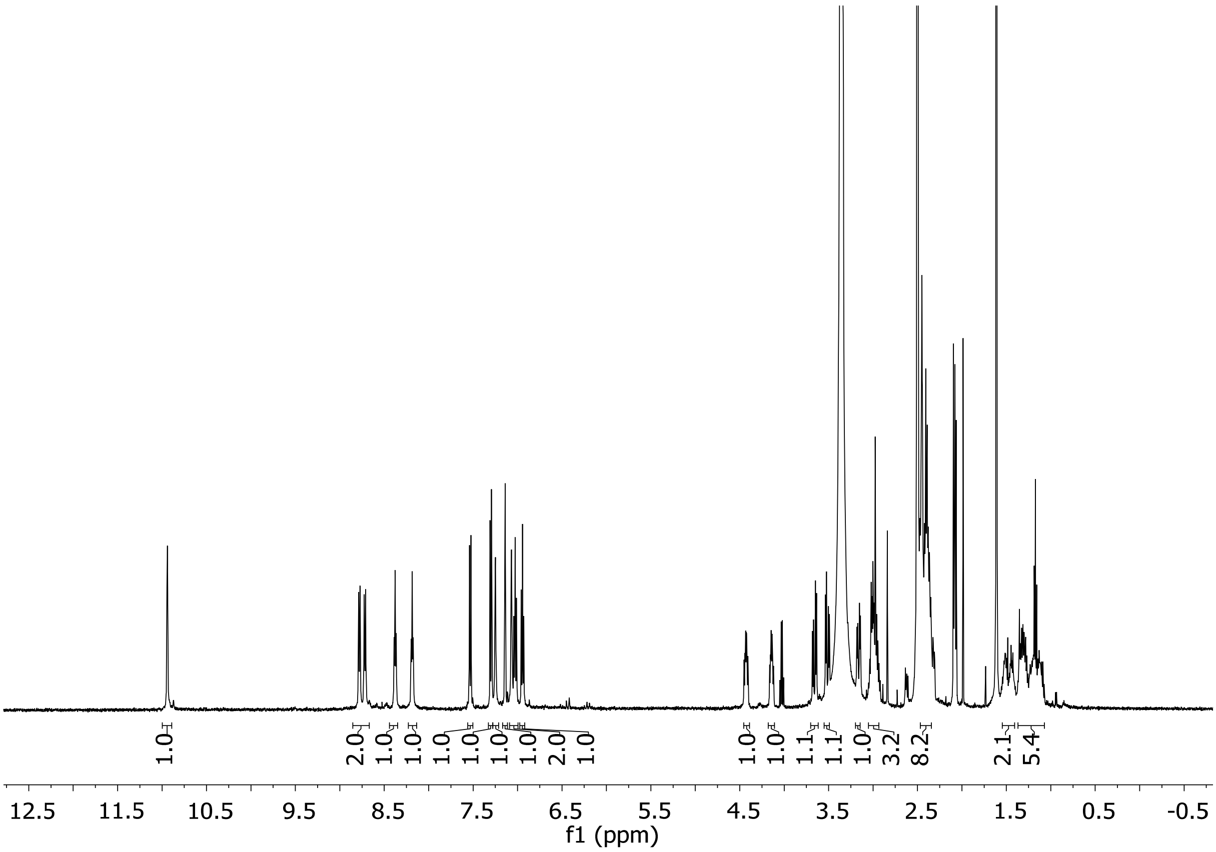

Figure S2. ^{1}H-NMR (DMSO-d_6, 500 MHz, 293 K) of compound **5**.

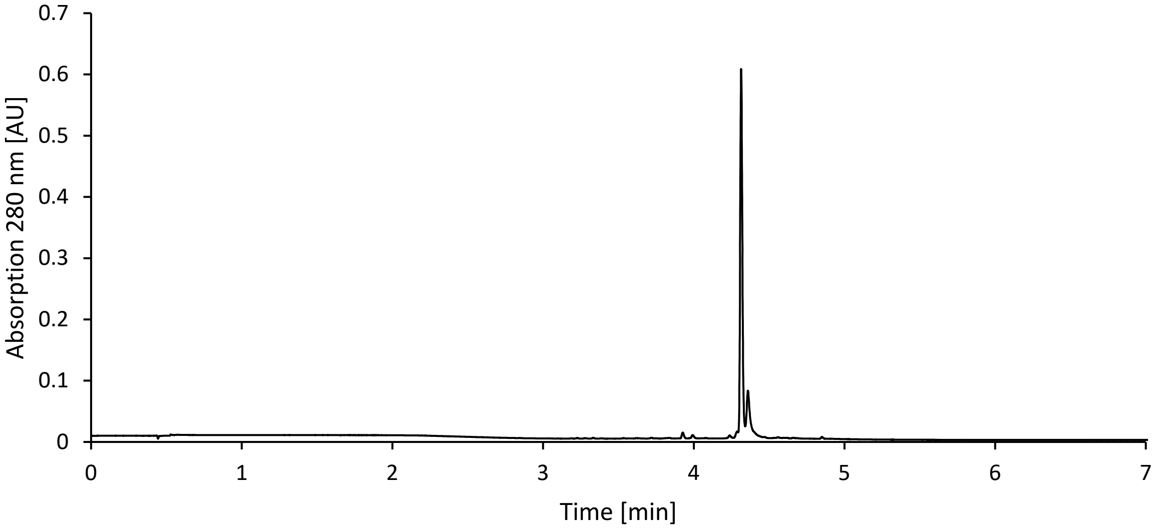

Figure S3. UPLC Chromatogram of compound **5**.

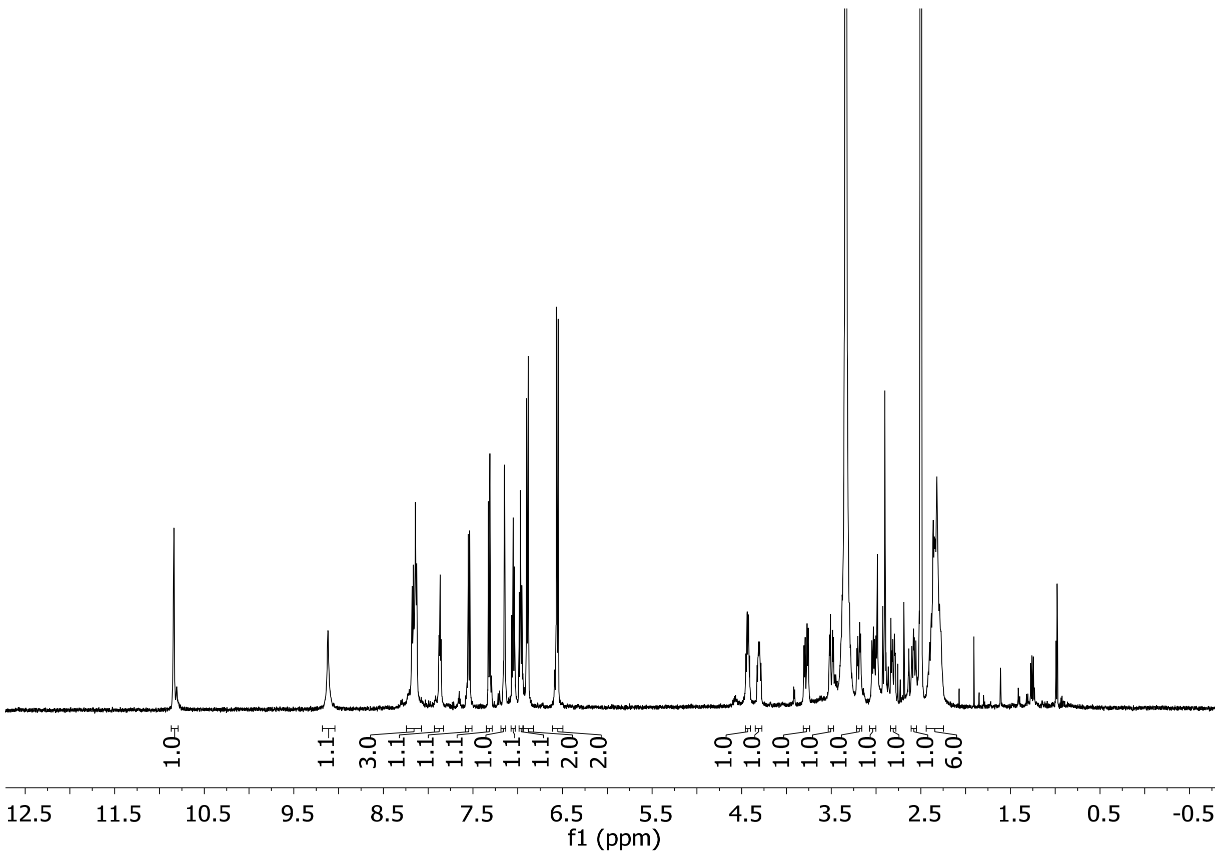

Figure S4. ^{1}H-NMR (DMSO-d_6, 500 MHz, 293 K) of compound **6**.

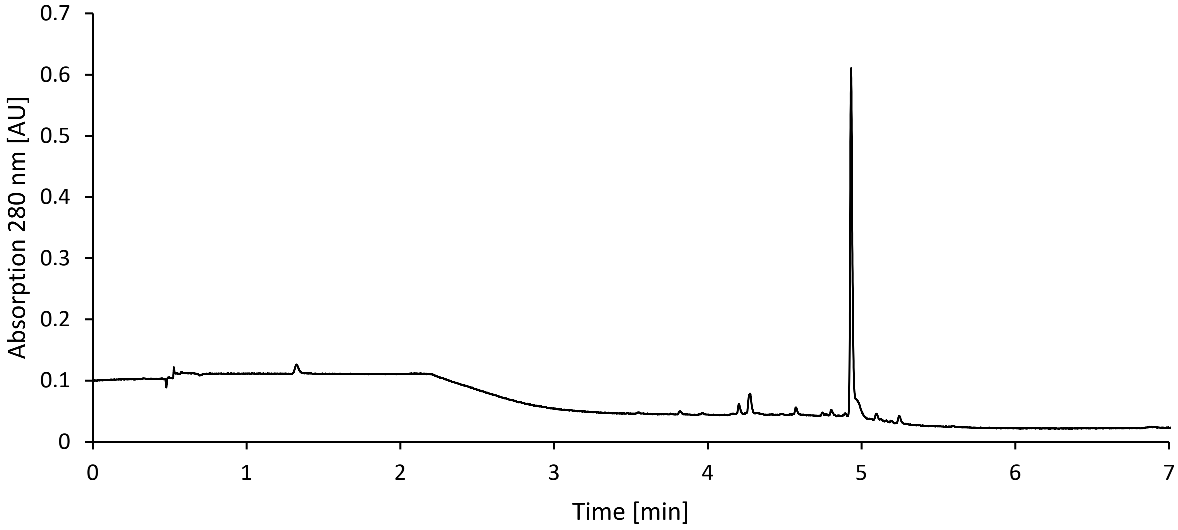

Figure S5. UPLC Chromatogram of compound **6**.

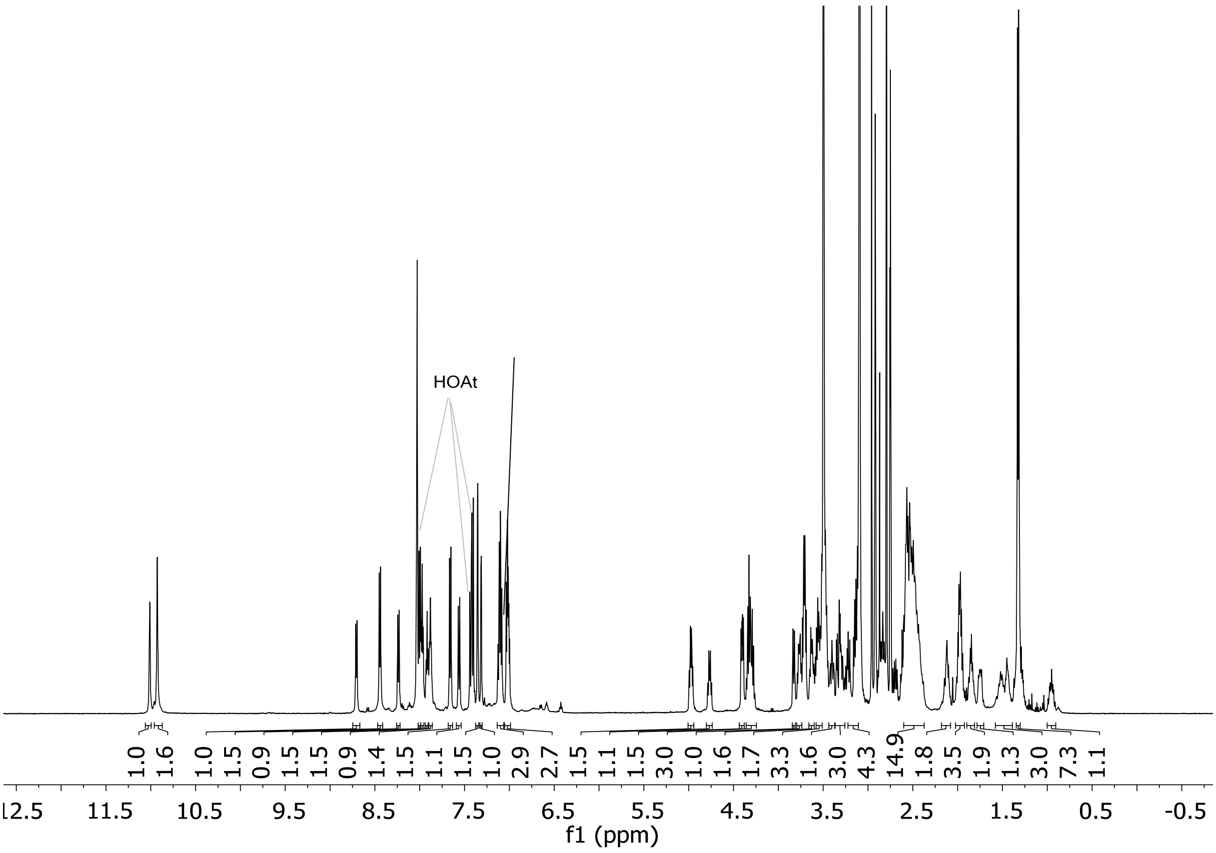

Figure S6. ^{1}H-NMR (DMSO-d_6, 500 MHz, 293 K) of compound **7.** HOAt impurity is indicated.

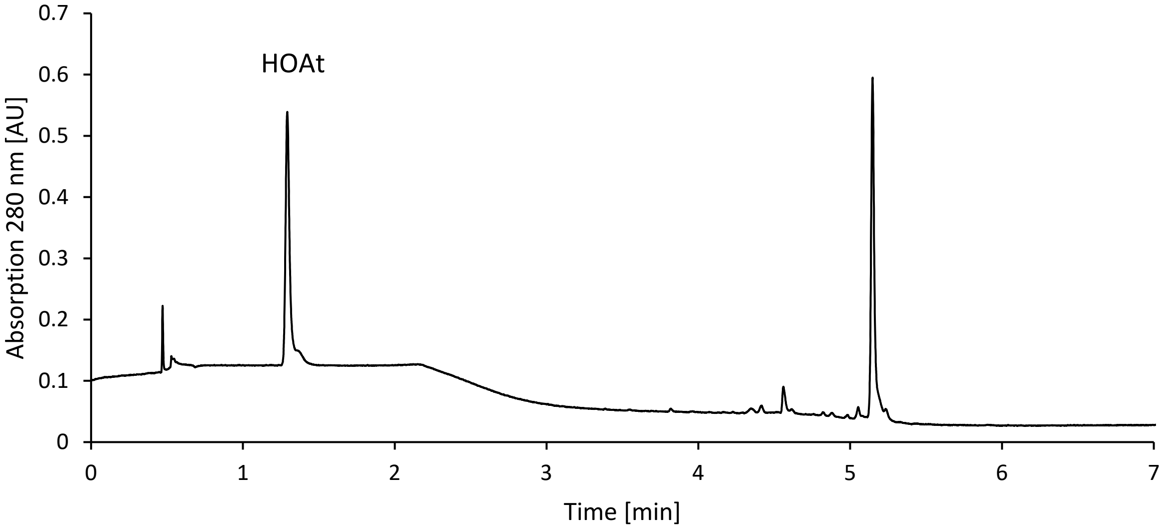

Figure S7. UPLC Chromatogram of compound **7**. HOAt impurity is indicated.

Experimental details for interference and deflection experiments

The interference and deflection experiments require ultrahigh vacuum to avoid collisional decoherence, with typical levels of 1-2 $\times 10^{-8}$ mbar during operation. During deflection experiments, compounds **1** and **2** were heated to 275-290° C in the thermal source, a temperature range which provided a reasonable balance between beam intensity and fragmentation. The molecules were ionized via electron impact bombardment, mass selected in an Extrel quadrupole (MAX-4000HT, 19 mm rods, 440 kHz operating frequency), and counted via a dynode-Channeltron assembly. The optical phase grating of the interferometer was supplied by a Coherent Verdi V20, with a power setting of 9 W for compound **1** and 8 W for compound **2**. Beam velocities were measured via cross-correlation with a pseudo-random sequence imprinted in the molecular beam via a mechanical chopper.[2]

The relative alignment of the three gratings is critical for high contrast interference, most importantly the grating roll angles (around the longitudinal beam axis) with respect to gravity and to each other, the inter-grating separation, and the yaw of the second grating.[3] The alignment is verified by optimizing the interference visibility of C_{60} to avoid wasting the limited tripeptide sample. The laser power of the second grating for the tripeptide experiments depends on the optical polarizability of the molecules at 532 nm, which is not known a priori. For this reason, a series of interference scans with varying power settings was performed to determine the optimal power setting, although the limited amount of synthesized material limited the accuracy of this procedure. Some visibility reduction is also expected due to the partial compensation of the velocity-dependent Coriolis force[4], since a roll alignment optimized for a faster C_{60} beam was employed for the tripeptide experiments.

For each G_3 position of an interference scan (e.g. those shown in Figure 3) a reference measurement with the electrode at 250 V was taken along with a measurement at a higher deflection voltage. This procedure limits the influence of phase drift on the order of the integration time; phase stability at LUMI is typically on the order of several nanometers per minute, so any effect of drift can be safely neglected. The reported phases shown in Figure 4 are obtained by subtracting the phase of the reference curves from that of the deflected curves. Further details on the geometry of the electrode and the LUMI experimental setup can be found in the references supplied in the main text.

References

[1] J. Schätti, U. Sezer, S. Pedalino, J.P. Cotter, M. Arndt, M. Mayor, V. Köhler, Tailoring the volatility and stability of oligopeptides, J. Mass Spectrom., 52 (2017) 550-556.
[2] G. Comsa, R. David, B.J. Schumacher, Magnetically suspended cross-correlation chopper in molecular beam-surface experiments, Rev. Sci. Instrum., 52 (1981) 789-796.
[3] K. Hornberger, S. Gerlich, H. Ulbricht, L. Hackermüller, S. Nimmrichter, I. Goldt, O. Boltalina, M. Arndt, Theory and experimental verification of Kapitza-Dirac-Talbot-Lau interferometry, New J. Phys., 11 (2009) 043032.
[4] Y.Y. Fein, F. Kialka, P. Geyer, S. Gerlich, M. Arndt, Coriolis compensation via gravity in a matter-wave interferometer, New J. Phys., (accepted, 2020).
